# Proteome‐Guided Drug Target Discovery for Periodontitis

**DOI:** 10.1111/jcpe.70032

**Published:** 2025-09-05

**Authors:** Zoheir Alayash, Sebastian‐Edgar Baumeister, Birte Holtfreter, Thomas Kocher, Hansjörg Baurecht, Benjamin Ehmke, Daniel Hagenfeld, Stefan Lars Reckelkamm, Michael Nolde

**Affiliations:** ^1^ Institute of Health Services Research in Dentistry University of Münster Münster Germany; ^2^ Department of Restorative Dentistry, Periodontology, Endodontology, and Preventive and Pediatric Dentistry University Medicine Greifswald Greifswald Germany; ^3^ Department of Epidemiology and Preventive Medicine University of Regensburg Regensburg Germany; ^4^ Clinic for Periodontology and Conservative Dentistry University of Münster Münster Germany

**Keywords:** drug discovery, genetics, periodontitis, proteome‐wide Mendelian randomisation analysis, target genes

## Abstract

**Background and Objective:**

Periodontitis is a chronic inflammatory disease driven by immune dysfunction and microbial imbalance. This study aims to identify circulating druggable proteins causally linked to the disease.

**Materials and Methods:**

We integrated proteomics data from deCODE genetics with periodontitis genome‐wide association studies (GWAS) from the Million Veteran Program to identify proteins associated with periodontitis. Findings were replicated using GWAS data from the Gene‐Lifestyle Interactions in Dental Endpoints consortium. Causal associations were validated using genetic and statistical methods, and the identified proteins were assessed for biological relevance and druggability.

**Results:**

Among the 2088 evaluated proteins, three showed robust evidence of causal association with periodontitis: FGF2 (fibroblast growth factor 2) (odds ratio [OR]: 1.06, 95% confidence interval [CI] 1.032–1.082), AZGP1 (zinc‐alpha‐2‐glycoprotein) (OR: 1.12, 95% CI 1.058–1.189) and BTC (betacellulin) (OR: 0.86, 95% CI 0.789–0.942). Replication analysis confirmed associations for 18 proteins, with 16 showing high colocalisation. Further evaluation of drug target databases revealed indirect links between the identified proteins and approved therapies for inflammatory conditions, suggesting potential therapeutic relevance.

**Conclusion:**

This study identifies three circulating proteins—FGF2, AZGP1 and BTC—as causally associated with periodontitis, highlighting their potential as therapeutic targets. These results provide a foundation for future research into targeted therapies for periodontitis.

## 1 Introduction

Periodontitis is a chronic inflammatory disease resulting from a complex interaction between an imbalanced host immune response and dysbiotic polymicrobial communities that colonise subgingival tooth surfaces (Lamont et al. 2018). Genetic predisposition can contribute to exaggerated inflammatory reaction, while environmental factors (such as stress and diet), behavioural risks (including smoking) and systemic conditions (such as diabetes and rheumatoid arthritis) can further impair immune function and increase susceptibility (Hajishengallis and Chavakis 2021; Loos and van Dyke 2020; Sczepanik et al. 2020; Zhao et al. 2023). An uncontrolled inflammatory response can lead to the destruction of periodontal tissues, and if untreated, may result in tooth loss, difficulty chewing, poor nutrition, aesthetic concerns, speech problems and diminished self‐confidence, ultimately impacting the overall quality of life (Liu, Liu, et al. 2024; Uy et al. 2022). Periodontal tissue destruction in periodontitis is largely due to the uncontrolled host inflammatory response in susceptible individuals (Hajishengallis and Korostoff 2017). In 2021, severe periodontitis affected more than 1 billion individuals worldwide with age‐standardised prevalence of 12.5% (Nascimento et al. 2024). Beyond its impact on oral health, periodontitis is linked to systemic conditions such as cardiovascular diseases, diabetes, rheumatoid arthritis and Alzheimer's disease (Hajishengallis and Chavakis 2021). These broader health implications highlight the need for effective management strategies to prevent disease progression and mitigate its systemic effects. As a major cause of tooth loss globally, periodontitis poses a significant public health challenge, with millions affected worldwide (Tonetti et al. 2017).

Periodontitis treatment typically begins with improving oral hygiene and controlling risk factors. This is followed by the professional removal of supragingival and subgingival biofilm and calculus, with adjunctive treatments considered in advanced cases. While systemic antibiotics remain the primary adjunctive therapy, emerging evidence suggests a promising future for novel therapeutic strategies (Sanz et al. 2020). Over the past five decades, our understanding of periodontitis has evolved significantly, particularly regarding the role of inflammatory cytokines in shaping the disease process (Hajishengallis and Korostoff 2017). The Page and Schroeder model offered a foundational framework for identifying key cellular players in periodontal disease (Page and Schroeder 1976). However, recent insights have highlighted the crosstalk between immune cells and their inflammatory cytokines. These cytokines are recognised as critical mediators, influencing leukocyte interactions, complement activation and osteoclastogenesis (Hajishengallis and Korostoff 2017). Despite these advances, significant knowledge gaps remain regarding the cytokine networks in periodontitis. Addressing these gaps will be essential for developing targeted therapeutic strategies.

Human proteins are fundamental to numerous biological processes and serve as targets for drug development (Zheng et al. 2020). Genetic studies have found that drugs targeting proteins with a genetically supported link to a disease are twice as likely to gain market approval (Nelson et al. 2015). In this context, Mendelian randomisation (MR) analysis has recently gained popularity in drug discovery and drug repurposing (Reay and Cairns 2021). MR is based on the instrumental variable (IV) approach that typically leverages single nucleotide polymorphisms (SNPs) from genome‐wide association studies (GWAS) to assess the causal relationship between traits. Compared with other observational study designs, MR reduces the bias that arises from confounding and reverse causation. Advances in high‐throughput genomic and proteomic technologies have enhanced MR‐based methodologies, facilitating the identification of promising therapeutic targets for health conditions (Chong et al. 2019; Wingo et al. 2021). To date, three studies have attempted to explore potential circulating proteins in connection to periodontitis through proteome‐wide MR analysis (Huoshen et al. 2025; Liu, Wang, et al. 2024; Zhan et al. 2024).

In our study, we performed a large‐scale proteome‐wide MR analysis to identify proteins causally associated with periodontitis, applying a novel and rigorous approach. Using summary statistics from a large‐scale protein quantitative trait loci (pQTL) study of 4719 circulating proteins in Icelandic individuals (Ferkingstad et al. 2021) and GWAS data for periodontitis from the Veterans Affairs Million Veteran Program (VA‐MVP), we aimed to gain genetic insights into protein targets involved in the pathogenesis of periodontitis. Rigorous validation, using periodontitis GWAS obtained from the Gene‐Lifestyle Interactions in Dental Endpoints (GLIDE) consortium, colocalisation and sensitivity analyses, was conducted to confirm causal associations and address potential confounding due to linkage disequilibrium (LD) or reverse causation. Furthermore, druggability assessments and protein–protein interaction (PPI) networks were employed to evaluate whether the proteins identified have been studied in other inflammatory conditions and to assess their therapeutic potential in periodontitis.

## 2 Material and Methods

### 2.1 Study Exposure

Summary‐level statistics of genetic associations with serum levels of 4719 circulating proteins were obtained from a pQTL study conducted in 35,559 Icelanders. The proteomic profiling was performed using a multiplexed, modified aptamer‐based binding assay (SOMAscan version 4.0), which enables high‐throughput measurement of protein concentrations. Protein levels were pre‐processed through rank‐inverse normal transformation, adjusting for age and sex. Comprehensive methodological details are available in the original publication (Ferkingstad et al. 2021).

### 2.2 Study Outcome

#### 2.2.1 Discovery Analysis

Summary statistics for periodontitis were obtained from the VA‐MVP, a national cohort launched in 2011 to investigate the interplay between genetics, lifestyle and military exposures with health and disease among US veterans (Gaziano et al. 2016). The cohort comprises 34,270 periodontitis cases and 396,445 controls of European ancestry (Verma et al. 2024). The MVP's robust infrastructure includes a biorepository with blood specimens for DNA isolation and genotyping, systematically linked with the VA electronic health record (VA‐EHR). This linkage provides access to comprehensive diagnostic information, including International Classification of Diseases ninth and tenth revisions (ICD‐9 and ICD‐10) codes, laboratory measurements and a detailed enrolment questionnaire (Gaziano et al. 2016). Clinical outcomes were defined using Phecodes, a sophisticated system of curated ICD code groupings that transform diagnostic codes into clinically meaningful phenotypes (Bastarache 2021). Case status for binary Phecodes was determined by the presence of two or more instances of corresponding Phecode‐mapped ICD‐9 Clinical Modification (CM) or ICD‐10 CM codes in the EHR. Conversely, control status was defined by the absence of these mapped ICD CM codes.

#### 2.2.2 Replication Analysis

Summary statistics for periodontitis were obtained from the GLIDE consortium, comprising seven cohort studies of European descent. The dataset encompasses 17,353 clinically diagnosed periodontitis cases and 28,210 controls. Periodontitis diagnosis was ascertained through multiple standardised methods: (i) the Centers for Disease Control and Prevention/American Academy of Periodontology classification (Roy C. Page and Eke 2007) and (ii) the Community Periodontal Index case definition. In less than 8% of the cases included, diagnosis status was reported by patients and not reassessed for the study (Shungin et al. 2019).

### 2.3 Instrument Selection

From the proteome GWAS, we selected cis‐pQTLs as genetic instruments. These cis‐pQTLs were specifically defined as SNPs situated within ±1 Mb of the transcription site of their corresponding protein‐coding genes. We identified SNPs significantly associated with protein levels (*p* < 5 × 10^−6^), applying a gene‐level significance threshold (Schmidt et al. 2020), and implemented LD clumping to retain only variants with minimal correlation (*r* ^2^ < 0.1). We evaluated instrument strength using both *R* ^2^ and *F*‐statistics (*F* ≥ 10) while also excluding rare genetic variants by only including SNPs with minor allele frequencies exceeding 1%.

### 2.4 MR Analysis

We conducted MR analyses using the ‘TwoSampleMR’ package in R 4.3.2. The causal effect, represented by the Wald ratio, was calculated as the instrument–outcome association divided by the instrument–exposure association. For proteins with two or more IVs, estimates were then obtained through a meta‐analysis using the inverse variance weighted (IVW) method. The causal estimate was expressed as the odds of periodontitis for each standard deviation (SD) increase in protein level. To address multiple testing, we controlled the false discovery rate (FDR) with the Benjamini–Hochberg (BH) procedure (Korthauer et al. 2019). With 2180 unique plasma proteins retained after rigorous selection and harmonisation, this provides a robust approach to managing potential false discoveries. In the replication analysis, we further investigated causal associations identified in the primary analysis using independent GWAS summary data, again controlling the FDR with BH.

To strengthen causal inference, we conducted several post hoc analyses. These included Steiger filtering to assess the directionality of associations, the HEIDI (Heterogeneity in Dependent Instruments) test for heterogeneity in dependent instruments, colocalisation analyses to evaluate shared causal variants and text‐mining to quantify association strength. We also assessed druggability and PPI clustering to explore therapeutic relevance. For methodological details, see Appendix A.

## 3 Results

### 3.1 Main Findings

**FIGURE 1:**
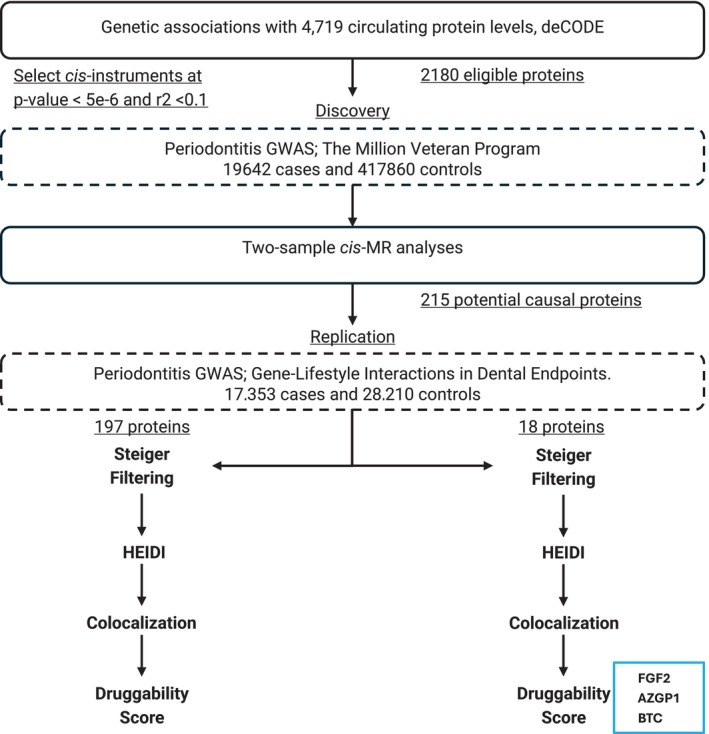
Overview of study design and results. This study used Mendelian randomisation (MR) to identify circulating proteins causally associated with periodontitis. Genetic instruments were derived from a proteome‐wide genome‐wide association study (GWAS) of 4719 proteins in 35,559 Icelanders. Summary statistics for periodontitis were obtained from the Million Veteran Program (discovery) and the Gene‐Lifestyle Interactions in Dental Endpoints consortium (replication). Three proteins—fibroblast growth factor 2 (FGF2), zinc‐alpha‐2‐glycoprotein (AZGP1) and betacellulin (BTC)—were robustly linked to periodontitis through multiple analytical layers, including replication, Steiger filtering, Heterogeneity in Dependent Instruments (HEIDI) and colocalisation analyses. Protein relevance was further supported by druggability assessments using Open Targets.

The study examined the MR associations between 2180 proteins with available index pQTL signals and periodontitis. An overview of the study design and summary of the results is presented in Figure 1. Three proteins were identified as key targets, supported by strong evidence in our analysis: FGF2 (fibroblast growth factor 2), AZGP1 (zinc‐alpha‐2‐glycoprotein) and BTC (betacellulin). For FGF2, a standard deviation increment in genetically predicted protein levels was associated with an odds ratio (OR) of 1.06 (95%, confidence interval [CI] 1.032–1.082). For AZGP1, we found an OR of 1.12 (95% CI 1.058–1.189) and for BTC, an OR of 0.86 (95% CI 0.789–0.942). Each of those proteins was validated through multiple methodological approaches, including replication analysis, Steiger filtering, HEIDI test and colocalisation analysis. The Open Targets association scores for these proteins were 0.57 for FGF2, 0.20 for AZGP1 and 0.15 for BTC, providing additional context for their potential biological relevance to periodontitis (Table 1).

**TABLE 1:** Mendelian randomization analysis and colocalisation of circulating proteins with periodontitis.

Gene	protein	*N*snps	OR	95% CI	*p*‐value after FDR adjustment	*p*‐value for HEIDI test	Steiger filtering	PPH4
FGF2	Fibroblast growth factor 2	102	1.06	1.032–1.082	0.0002	0.8487	TRUE	100
AZGP1	Zinc‐alpha‐2‐glycoprotein	28	1.12	1.058–1.189	0.0037	0.5678	TRUE	99.5
BTC	Betacellulin	11	0.86	0.789–0.942	0.0202	0.4899	TRUE	99.3

*Note*: The OR was scaled to one standard deviation increase in genetically predicted circulating protein levels.

Abbreviations: CI, confidence interval; FDR, false discovery rate; HEIDI, Heterogeneity in Dependent Instruments; Nsnps, number of single nucleotide polymorphisms; OR, odds ratio; PPH4, posterior probability of hypothesis 4 (both traits are associated with a causal variant).

### 3.2 Two‐Sample MR

Using the VA‐MVP GWAS for two‐sample cis‐MR, 215 protein–periodontitis associations were observed. Replication analysis using the GLIDE GWAS confirmed associations for 18 of those proteins (Table S1). To account for multiple testing, we applied the FDR approach to the results of both analyses. To give a full picture of our results for future investigations, the remaining proteins that were not replicated via the replication analysis (197 proteins) were further assessed using Steiger filtering, HEIDI test and colocalisation analysis (Figure 1).

### 3.3 Steiger Filtering and HEIDI Test

Steiger filtering was applied to ensure directionality among the 18 replicated proteins, with all passing this test. Additionally, all 18 proteins passed the HEIDI test for pleiotropy (Table S1). Among the 197 proteins that could not be replicated, no evidence of reverse causation was found, but 17 associations failed the HEIDI test due to potential pleiotropy (Table S2).

### 3.4 Colocalisation

Colocalisation analysis was conducted to assess whether the associations between circulating proteins and periodontitis shared causal variants. Of the 18 proteins validated through replication, 16 demonstrated high colocalisation support with posterior probabilities for shared causal variants PPH4 ≥ 0.9. However, PARK7 and CD163 showed lower posterior probabilities of 0.09 and 0.14, respectively (Table S1). Additionally, 169 proteins from the non‐replicated group exhibited high colocalisation support (Table S2).

### 3.5 Association With Periodontitis

We searched the proteins in Open Targets to assess their association with periodontitis. Six proteins were identified as being associated with periodontitis, with ‘Europepmc’ association scores based on text mining ranging from 0.02 to 0.57 (Table S1), supporting the relevance of further investigating these associations. For reference, these correspond to a relatively moderate rank of 204 (FGF2), 645 (AZGP1) and 815 (BTC) among 3242 proteins that have shown any association with periodontitis. Among proteins that were not replicated but were further analysed, association scores ranging from 0.01 to 0.97 were retrieved for 68 proteins (Table S2).

### 3.6 Druggability of Identified Proteins

The druggability of proteins identified through MR analysis was evaluated using drug databases. None of the proteins could be identified as direct drug targets for periodontitis. However, several proteins were linked to treatments for other inflammatory conditions. For example, TNF‐α inhibitors such as certolizumab pegol, etanercept and adalimumab, as well as the α‐1 proteinase inhibitor (a leukocyte elastase inhibitor in Phase 1 trials), are approved for inflammation‐related diseases. Given the inflammatory nature of periodontitis, these findings suggest a potential indirect relevance, although direct evidence or clinical trials are necessary to confirm any therapeutic implications.

### 3.7 PPI Networks

**FIGURE 2:**
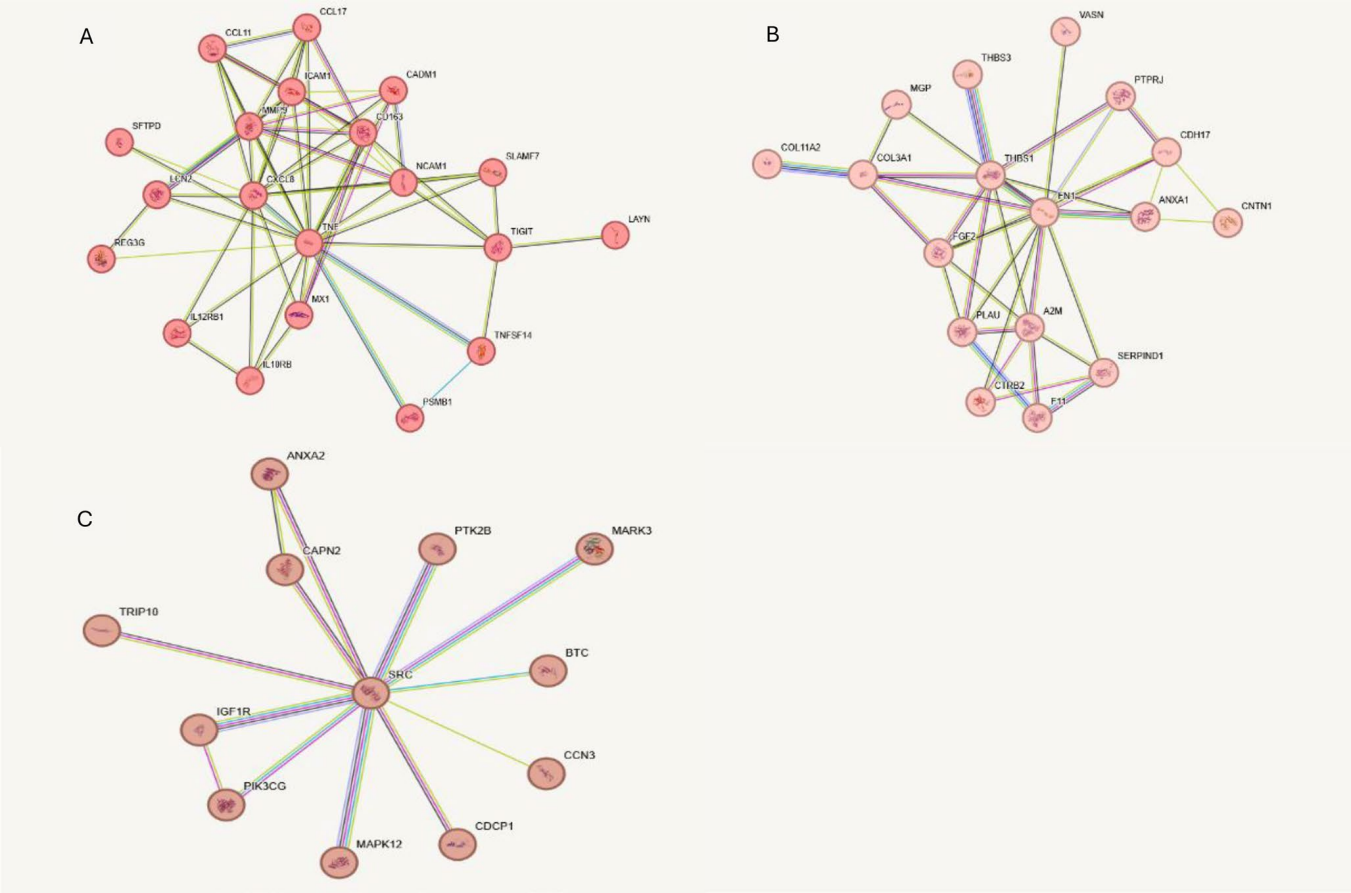
Network analysis of 215 potential drug target genes using the STRING database. Three main protein clusters were identified: (A) the largest cluster (20 proteins) associated with interleukin (IL)‐17, IL‐4 and IL‐13 signalling pathways; (B) a cluster of 17 proteins including FGF2; and (C) a cluster of 12 proteins including BTC.

Two‐hundred and fifteen potential drug target genes were loaded into the STRING database for network creation. Three main clusters containing 20, 17 and 12 proteins were identified. The first and largest cluster, encompassing 20 proteins, represents the signalling pathways associated with interleukin (IL)‐17, IL‐4 and IL‐13 (Figure 2A). While these ILs themselves are not shown in the figure, their downstream signalling components and related functional pathways are represented. The second cluster includes FGF2 together with 16 other proteins identified in our discovery analysis (Figure 2B). The third main cluster includes BTC and 11 other proteins. The rest of the proteins were distributed over smaller clusters (Figure 2C).

## 4 Discussion

In our comprehensive proteome‐wide search for drug targets, we initially filtered 2088 circulating proteins and examined their associations with periodontitis risk and identified 215 significant associations. Among these proteins, 18 were found to be consistently associated with periodontitis using an alternative outcome GWAS for replication (Table S1). Further scrutiny examining colocalisation and directionality, and considering known protein interactions and prior evidence, suggested FGF2, AZGP1 and BTC as key targets for periodontitis. These targets were supported by strong evidence and passed all the rigorous sensitivity tests.

### 4.1 FGF2

FGF2 was identified as the most promising target with strong evidence from all aspects. It showed the strongest previously known correlation with periodontitis based on Open Targets. FGFs represent a diverse group of growth factors essential for angiogenesis, wound healing and tissue regeneration. Among these, FGF‐2 has emerged as one of the most extensively studied, primarily due to its ability to bind to heparin and exhibit potent mitogenic and angiogenic properties (Kao et al. 2009; Murakami 2011). FGF‐2 has shown bone‐promoting effects through the differentiation of osteoprogenitor cells while stimulating the proliferation and migration of periodontal ligament cells. These properties have positioned FGF‐2 as a key therapeutic candidate for the regeneration of periodontal tissues (Mayahara et al. 1993; Murakami et al. 2003; Nakamura et al. 1995; Takayama et al. 2001). Notably, recombinant human FGF‐2 (rhFGF‐2) is already approved in Japan for periodontitis therapy (Hui et al. 2018; Saito et al. 2019).

Five randomised controlled trials (RCTs) have evaluated its efficacy for periodontal defect treatment (Cochran et al. 2016; Kitamura et al. 2008; Kitamura et al. 2011; Kitamura et al. 2016; Seshima et al. 2022). When administered as 200 μL of a hydroxypropylcellulose‐based gel during flap surgery, rhFGF‐2 consistently showed significant improvements in bone regeneration. The 0.3% dose was particularly effective, increasing alveolar bone by up to 58.62% compared to controls (Kitamura et al. 2008). In another trial, this dose significantly enhanced the bone fill percentage at 36 weeks, highlighting its therapeutic potential and safety (Kitamura et al. 2011). When combined with β‐tricalcium phosphate, rhFGF‐2 further improved clinical attachment gains (≥ 1.5 mm) and linear bone growth (≥ 2.5 mm), achieving success rates of 71% in the 0.3% and 0.4% groups compared to 45% in the 0.1% and control groups, with bone fill reaching up to 75% (Cochran et al. 2016). In addition, Phase III trials confirmed that rhFGF‐2 significantly increased bone fill (37.1% vs. 21.6%) and demonstrated substantial linear alveolar bone growth (1.93 mm). A study comparing rhFGF‐2 with an enamel matrix derivative showed that rhFGF‐2 provided superior outcomes (1.93 vs. 1.36 mm), further supporting its effectiveness. Importantly, no safety concerns were reported in either trial, reinforcing the potential of rhFGF‐2 as a reliable and effective therapy for periodontal tissue regeneration (Masahiro Kitamura et al. 2016; Seshima et al. 2022).

### 4.2 AZGP1

Similar to FGF2, but with less prior evidence in relation to periodontitis, AZGP1, a class I major histocompatibility complex domain glycoprotein, showed strong evidence at all phases of our analysis. Previously, elevated levels of AZGP1 have been reported in the unstimulated saliva of patients with periodontitis (Wu et al. 2009). AZGP1 emerges as a critical mediator linking macrophage dysfunction, inflammation and periodontal tissue destruction, positioning it as a potential therapeutic target for the treatment of periodontitis. Yang et al. (2024) found that the AZGP1 expression was significantly increased in macrophages stimulated by lipopolysaccharides and gingival tissues from both patients and mice with periodontitis. Besides, overexpression of AZGP1 exacerbated periodontal inflammation and alveolar bone loss, whereas its knock‐out attenuated periodontal inflammation and alveolar bone loss. AZGP1 was primarily colocalised with macrophages, by promoting M1 polarisation and pyroptosis through the NLR family pyrin domain containing 3 (NLRP3)/caspase‐1 pathway in periodontitis tissues, thus blocking the NLRP3/caspase‐1 pathway with NLRP3 or caspase‐1 inhibitor reversed macrophage M1 polarisation and pyroptosis induced by AZGP1 overexpression. The lack of further research on the relationship between AZGP1 and periodontitis makes this an interesting and novel drug target that warrants future examination.

### 4.3 BTC

In addition, our analysis identified BTC, providing new evidence to support the findings of a previous study investigating the expression and role of BTC, a member of the epidermal growth factor family, in chronic periodontitis (Ugale et al. 2015). Results there showed that BTC expression was significantly higher in chronic periodontitis tissues compared to healthy controls. A positive correlation was observed between BTC expression and clinical parameters, particularly probing depth, suggesting an association with disease severity. BTC likely contributes to tissue destruction by inducing the production of inflammatory mediators such as matrix metalloproteinase‐9 (MMP‐9) and prostaglandin E2, which are implicated in the degradation of extracellular matrix and bone resorption in periodontitis (Ugale et al. 2015).

### 4.4 Further Prominent Protein Targets

In addition to the main protein targets highlighted by our study, we identified several other proteins with a lower degree of evidence but with potential relevance to periodontitis, as suggested by existing literature, whose failure to pass the replication analysis might be due to the lack of statistical power. Among these, MMP‐9 and tumour necrosis factor (TNF) have been extensively studied for their involvement in the pathogenesis of periodontal disease. In our analysis, MR findings revealed associations of MMP‐9 and TNF with periodontitis. Furthermore, colocalisation analysis indicated that these proteins are likely to share genetic loci with periodontitis.

The accessibility of proteome‐wide studies, along with the availability of novel and robust statistical methods to assess causality between proteins and disease outcomes, has created a fertile ground for scientists to accelerate drug discovery. To date, three other studies have looked for drug targets for periodontitis across the human proteome. The first study found associations between periodontitis and three genes: C‐X‐C motif chemokine ligand 10 (*CXCL10*), myosin IIIB (*MYO3B*) and signalling lymphocytic activation molecule family member 1 (*SLAMF1*) (Liu, Wang, et al. 2024). In our study, *MYO3B* was not reported in the deCODE GWAS, and *SLAMF1* was reported but we could not identify valid IVs. *CXCL10* was not significant in our discovery analysis. Our study had six IVs for *CXCL10*, compared to three IVs obtained by Liu et al., and used different IV selection criteria, as well as different datasets for both exposure and disease. The second proteome‐wide association study found that genetically predicted circulating levels of von Willebrand factor A domain‐containing 1 and growth differentiation factor 15 were associated with the disease (Zhan et al. 2024). Our discovery analysis identified no evidence of association between these targets and periodontitis. Finally, a previous study reported an association between the heme‐binding protein 1 and the angiopoietin‐1 receptor with periodontitis (Huoshen et al. 2025); however, our findings did not support this association after applying multiple testing correction.

Our study explored potential drug targets for periodontitis and identified several druggable genes associated with the condition. However, these did not include previously reported MR findings, such as complement components, IL‐17, IL‐6 receptor and TNF receptor 1 (Alayash et al. 2023; Alayash et al. 2024b; Alayash et al. 2024a; Baumeister et al. 2023; Nolde et al. 2023). This discrepancy may be due to our stringent analysis pipeline, which could obscure some potential druggable targets.

A major strength of our study lies in the application of protein drug target MR. To enhance the validity of our instruments and reduce the likelihood of horizontal pleiotropy, we selected genetic variants located near the encoding gene and associated with circulating protein levels (Holmes et al. 2021). This study goes beyond prior attempts because we systematically examined the associations between plasma protein biomarkers and periodontitis risk by employing a strict paradigm that incorporates MR study design with the advantages of large sample sizes and minimal risk of reverse causation and confounding bias. We combined a discovery analysis, which, for the first time, utilised the new and so far the largest VA‐MVP GWAS on periodontitis with a replication in the well‐established GLIDE GWAS. The consistency of results among multiple rigorous analyses confirmed the robustness of the study findings. Additional evidence from PPI and druggability evaluation provided insights into the potential effect of candidate proteins on periodontitis and further prioritised druggable targets. The lack of drug information on several proteins that were identified by our analysis prevents us from recommending them for instant further examination, but these proteins still make up a reservoir of promising new causative pathways in the pathogenesis of periodontitis. Nevertheless, several limitations of this study should also be considered. First, the current analysis was restricted to European populations. The generalisation of these findings to other ancestries needs to be further confirmed (Burgess et al. 2019). Second, we assessed the role of serum proteins in periodontitis but could not estimate the levels of relevant proteins in other tissue, such as the oral tissue. Assessing the role of protein levels from the oral tissue may provide more insight into disease pathogenesis. Third, Phecodes improve accuracy, but case definitions used in the initial discovery analysis were not clinically validated, which may introduce some misclassifications or diagnostic uncertainties. Our analysis, however, did not rely solely on this data, but also included an analysis of data with clinically diagnosed periodontitis. Finally, MR reflects lifelong genetic predisposition and may not directly translate to the impact of short‐term interventions. Therefore, our findings support a potential causal role of FGF2, AZGP1 and BTC in the aetiology of periodontitis, rather than quantifying the precise clinical magnitude of their effects (Woolf and Burgess 2025).

## 5 Conclusion

Our study identified several proteins that were associated with periodontitis risk and provided new insights into the aetiology of periodontitis and potential targets for the development of therapeutic drugs. Further research is needed to validate these targets and explore their clinical efficacy.

## Author Contributions

Conception and design: Z.A., S.E.B., M.N., S.L.R. Development of methodology: Z.A., S.E.B., H.B., S.L.R., M.N. Analysis and interpretation of data (e.g., statistical analysis, biostatistics, computational analysis): Z.A., S.E.B., M.N., S.L.R. Administrative, technical or material support (i.e., reporting or organising data, constructing databases): Z.A. All authors contributed to writing, review and/or revision of the manuscript.

## Ethics Statement

All analyses were based on publicly available summary statistics without accessing individual‐level data; hence, ethical approval was not required. The included GWAS received informed consent from the study participants and were approved by pertinent local ethical review boards.

## Conflicts of Interest

The authors declare no conflicts of interest.

## Supporting information


**Table S1:** Mendelian randomisation analysis and colocalisation of circulating –s with periodontitis.
**Table S2:** Mendelian randomisation analysis and colocalisation of circulating proteins with periodontitis.

## Data Availability

The periodontitis GWAS summary data from MVP are available at https://phenomics.va.ornl.gov/web/ and from GLIDE at https://data.bris.ac.uk/data/dataset/2j2rqgzedxlq02oqbb4vmycnc2. The proteomic GWAS summary data are available at https://download.decode.is/form/folder/proteomics.

## Appendix A

### Steiger Filtering/Reverse Causation

The Steiger filtering method was applied to MR associations that passed the multiple‐testing threshold to assess the potential distortion of results by reverse causality. This method evaluates whether the direction of effect aligns with the hypothesised causal pathway, from exposure to outcome (Hemani et al. 2017). Results are categorised as follows: ‘true’ indicates that the effect direction is consistent with exposure influencing the outcome (*p* > 0.05), while ‘false’ indicates evidence of reverse causality.

### Heterogeneity in Dependent Instruments (HEIDI) Test

HEIDI specifically tests whether the observed association is due to pleiotropy or linkage disequilibrium. A *p*‐value < 0.05 in the HEIDI test indicates that the association is likely due to pleiotropy, meaning that a single genetic variant influences both traits independently (Zhu et al. 2016).

### Colocalisation Analysis: Bayesian and SuSiE

The aim of this analysis was to assess whether two signals from the protein and periodontitis GWAS were consistent with shared causal variants, thereby distinguishing true causal relationships from confounding due to LD. We estimated the posterior probability of each genomic locus containing variants affecting both traits.

To achieve this, we employed two algorithms: the Bayesian statistical procedure, and the sum of single effects (SuSiE) regression framework. The Bayesian method assumes the presence of a single shared causal SNP (Giambartolomei et al. 2014). However, in reality, genetic loci may contain multiple causal SNPs. To address this, we applied SuSiE, which allows for multiple causal variants within a specified gene region (Wallace 2021).

For each protein, we included SNPs within ±1 Mb of the gene locus. The colocalisation analyses used the following prior probabilities: *p*1 = 1 × 10^−4^ (prior probability that an SNP is associated with the protein), *p*2 = 1 × 10^−4^ (prior probability that an SNP is associated with periodontitis) and *p*12 = 1 × 10^−5^ (prior probability an SNP is associated with both the protein and periodontitis). Proteins were considered to have strong evidence of colocalisation if the posterior probability for shared causal variant/s (PPH4) ≥ 90%, determined by at least one algorithm (Giambartolomei et al. 2014).

### Association Scores Based on Text Mining and Druggability Evaluation

Based on multiple sources of evidence, we obtained an association score to quantify the relationship between periodontitis and the targeted protein using Open Targets (https://www.opentargets.org/). The association score integrates information from various data sources to provide a nuanced assessment of the protein's potential therapeutic relevance. It ranges between 0 and 1, with higher scores indicating stronger evidence of association between the protein and the disease. The score is calculated by combining multiple types of evidence, including genetic associations, somatic mutations, differential expression, literature mining and known drug interactions. Each type of evidence is weighted and integrated to create a composite score. The score allows researchers to prioritise potential therapeutic targets by providing a systematic, data‐driven approach to assess a protein's relevance regarding a disease beyond traditional single‐source assessments (Carvalho‐Silva et al. 2019). In addition, we further assessed whether the proteins that were identified in our discovery analysis can serve as potential therapeutic targets by searching the interactions between these proteins and drugs using Open Targets.

### Protein–Protein Interaction (PPI) and Druggability Evaluation

To explore the potential interactions between the identified proteins, a PPI network was constructed using the STRING database version 11.5 (https://string‐db.org/), with only interactions of medium or higher confidence (> 0.4) presented. The confidence score represents the approximate probability that a predicted interaction exists, based on data sources such as text mining, experiments, databases, co‐expression, neighbourhood, gene fusion and co‐occurrence. To simplify things, we excluded nodes (proteins) that lacked connections with other nodes. To identify biologically plausible pathways, clustering was performed using the Markov clustering algorithm (MCL) with an inflation factor set at 3. MCL was selected over other clustering methods because of its robustness in identifying dense sub‐networks (clusters) within large, complex datasets. Unlike alternative algorithms, MCL effectively simulates stochastic flows across the network, allowing it to differentiate tightly connected regions, which is particularly useful for capturing functional modules in biological systems (Brohée and van Helden 2006; Shih and Parthasarathy 2012).

The inflation factor in MCL amplifies probabilities of strong connections and suppresses weaker ones, thus controlling the size and the number of clusters with higher values of the factor yielding smaller, more distinct clusters and lower values producing fewer, larger clusters. The choice of an inflation factor of 3 was guided by its proven effectiveness in balancing sensitivity and predictive accuracy (Brohée and van Helden 2006). Studies indicate that an intermediate value such as three provides a robust trade‐off, capturing biologically meaningful clusters without over‐fragmentation or loss of specificity.
